# Supplementary figures and images for: Algorithmically Reconstructed Molecular Pathways as the New Generation of Prognostic Molecular Biomarkers in Human Solid Cancers
Source: Proteomes. 2023 Aug 25;11(3):26. doi: 10.3390/proteomes11030026 (PMC10535530; doi:10.3390/proteomes11030026)

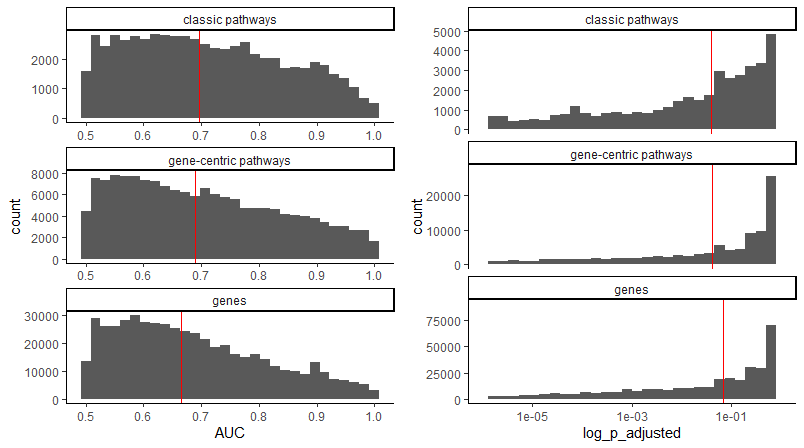

Supplement: Supplementary file 1 [file proteomes-11-00026-s001.zip › Supplementary_Figure__S1.png]
